# A Comprehensive Literature Review on the Management of Distal Deep Vein Thrombosis

**DOI:** 10.7759/cureus.8048

**Published:** 2020-05-10

**Authors:** Sohaip Kabashneh, Vijendra Singh, Samer Alkassis

**Affiliations:** 1 Internal Medicine, Wayne State University - Detroit Medical Center, Detroit, USA; 2 Hematology and Oncology, Karmanos Cancer Institute, Detroit, USA

**Keywords:** deep vein thrombosis (dvt), pulmonary embolism (pe), venous thromboembolism (vte)

## Abstract

Deep vein thrombosis (DVT) is a relatively common clinical entity with significant morbidity and mortality. Acute pulmonary embolism (PE) is the most significant complication of DVT and warrants immediate attention. The location of the DVT has a substantial impact on its ability to break off and travel to the pulmonary vasculature, causing a PE. Proximal DVT is more likely to cause a PE than a distal DVT. The widely accepted management for proximal DVT is anticoagulation. However, the management of distal DVT is unclear. This review article discusses factors that increase the risk of PE in patients with distal DVT, guidance on how to categorize patients into high and low-risk categories, and the recommended management for each category.

## Introduction and background

Venous thromboembolism (VTE), including deep vein thrombosis (DVT) and pulmonary embolism (PE), is a relatively common clinical entity with significant morbidity and mortality. The annual incidence of VTE in the United States (US) is estimated to be somewhere between 1-2 per 1,000 of the population [1-3]. Every year, about 2,000,000 new cases of DVT are diagnosed within the US [4].

The most serious complication of DVT is PE; the estimated 30-day case fatality rate of PE is 3.9% [5]. Annually, between 60,000 and 300,000 people die from PE in the US alone [4, 6-7]. PE has a higher in-hospital mortality rate than myocardial infarction.

The location of a DVT significantly affects the chances of embolization and eventually blocking a pulmonary vessel. A proximal DVT that is located in the popliteal, femoral or iliac veins is far more likely to embolize when compared to a distal DVT that is located below the knee and is confined to the calf veins (peroneal, posterior, anterior tibial, and muscular veins) without a proximal component [8]. 

A particularly challenging situation arises when a patient is diagnosed with an isolated distal DVT. Due to its low risk of embolization, the use of anticoagulation in this scenario is not clear. The purpose of this review is to highlight the current literature available to help guide physicians about the management of an isolated distal DVT.

## Review

Management of DVT depends on its location, whether it is proximal or distal. For patients with proximal DVT (PDVT), the widely accepted management is anticoagulant therapy for all patients (unless there is a contraindication) as it has a survival advantage with a decreased rate of recurrence [9]. Unlike PDVT, the management of distal DVT (DDVT) is controversial.

Chances of developing a PE or PDVT in patients with DDVT

Because the most serious complication of DVT is PE, understanding the natural history of DDVT, including isolated soleal or gastrocnemius vein thrombosis (ISGVT) and the chances of embolization, is key when deciding on the treatment. In an attempt to analyze this issue, Brateanu et al. conducted a study to evaluate the possible propagation of isolated DDVT to PDVT or PE [10]. Four hundred fifty patients with isolated distal DDVT were studied, and all the ultrasounds, chest ventilation/perfusion, and computed tomography scans ordered within three months after the initial DDVT were reviewed to determine the incidence of PDVT and/or PE. The conclusion was 22 (4.8%) patients developed PDVT, seven (1.5%) patients developed PE, and one patient developed both PDVT and PE. Two factors that were associated with thromboembolic complications were inpatient status and age. Outpatients were at low risk of developing PDVT/PE. Inpatients aged ≥ 60 years were at high risk. Inpatients aged < 60 were at intermediate risk.

MacDonald et al. also conducted a study on ISGVT propagation [11]. One hundred and thirty-five limbs with ISGVT were studied over three months. Twenty-two of the 135 limbs with ISGVT (16.3%) extended to the adjacent tibial or peroneal veins within the three month study period; the majority (90.9%) occurred within the first two weeks after the initial diagnosis [11]. Of the 22 limbs with ISGVT propagation, only four limbs (2.9%) extended up to the level of the popliteal vein. Of note, no ISGVT propagated proximally to the popliteal [11].

Both studies demonstrated a very low risk of DDVT embolization to PE [10-11]. Brateanu et al. revealed only 1.5% PE risk with DDVT, while MacDonald et al. revealed no ISGVT propagation above the popliteal vein.

The lack of significant benefit of anticoagulation in patients with DDVT

To establish the benefit of anticoagulation treatment for patients with DDVT, Sales et al. studied 141 patients with ISGVT, 76 received anticoagulation, while 65 patients did not [12]. Results from multivariate logistic regression showed that anticoagulation did not have a significant impact on propagation with an odds ratio of 1.28 (95% confidence interval: 0.55 - 3.01; P = .57). Thus, the study concluded a lack of efficacy of anticoagulation in ISGVT.

The Brateanu et al. study showed that the treatment of the isolated DDVT with therapeutic anticoagulation was associated with a lower risk of a PDVT and/or PE, but the association did not reach statistical significance [10]. Both studies concluded a lack of statistically significant benefit of anticoagulation in patients with DDVT [10, 12].

Treatment approach

In this article, we will discuss the guidelines adopted by the American College of Chest Physicians (ACCP) [13] and another approach suggested by Brateanu et al. [10].

ACCP Approach

Patients with isolated DDVT with one or more of the following risk factors should receive anticoagulant therapy for at least three months (Grade 1B). Those risk factors include:

1) Positive D-dimer

2) Thrombus more than 5 cm in length or in multiple veins

3) Thrombus in close proximity to proximal (popliteal) vein

4) Active cancer or immobility

However, in patients without risk factors for extension to PDVT and PE, and in patients with thrombi involving the muscular (soleus, gastrocnemius) veins alone, there is a weak recommendation for no anticoagulant therapy and serial imaging of the legs over the next two weeks (Grade 2C) [14].

*Brateanu* *et al. Approach*

Based on settings in which DDVT was diagnosed (inpatient vs. outpatient) and age, patients are categorized in low-risk, intermediate-risk, and high-risk categories. This classification is predictive of subsequent thromboembolic complications. For patients at high risk of subsequent thromboembolism (i.e., inpatients ages ≥ 60 years), anticoagulation may be prudent. In contrast, for patients at low risk (i.e., outpatients with age < 60 years), no treatment would appear to be necessary. For the remaining patients, serial ultrasounds may be reasonable, at least in the first month, when the risk is highest [10].

Distal DVT in trauma patients

Trauma patients are at higher risk for VTE due to immobilization. Olson et al. conducted a study on trauma patients with below-knee DVT (BKDVT) and/or above knee DVT (AKDVT) and found out that 12.9% of patients with BKDVT progressed to AKDVT or PE [15]. In the study population, BKDVT progressed to AKDVT or PE in one of eight patients. Actually, in this study, BKDVT was associated with a higher rate of PE compared with AKDVT, which was likely secondary to the treatment of AKDVT.

The study concluded that BKDVT should not be ignored in trauma patients and suggested that therapeutic anticoagulation should be considered in trauma patients with BKDVT [15].

## Conclusions

Management of DDVT has always been a dilemma among health care providers. This review of the literature shows that patients with DDVT are at very low risk of developing PE, and anticoagulation treatment is warranted only in high-risk patients. One approach uses inpatient status and age ≥ 60 years to stratify patients into either high, intermediate, or low-risk groups and manage accordingly. Another approach is to use comorbidities, immobility, D-dimer, and thrombus size to guide the treatment. However, trauma patients with DDVT should always be managed with anticoagulation regardless of their risk factors. 
